# Selective photocatalytic reduction of CO_2_ to methanol in CuO-loaded NaTaO_3_ nanocubes in isopropanol

**DOI:** 10.3762/bjnano.7.69

**Published:** 2016-06-01

**Authors:** Tianyu Xiang, Feng Xin, Jingshuai Chen, Yuwen Wang, Xiaohong Yin, Xiao Shao

**Affiliations:** 1 School of Chemical Engineering and Technology, Tianjin University, Tianjin 300072, China; 2 Collaborative Innovation Center of Chemical Science and Engineering (Tianjin), Tianjin 300072, China; 3 School of Chemistry and Chemical Engineering, Tianjin University of Technology, Tianjin 300384, China

**Keywords:** CO_2_ reduction, CuO loading, isopropanol, NaTaO_3_ nanocubes, photocatalysis

## Abstract

A series of NaTaO_3_ photocatalysts were prepared with Ta_2_O_5_ and NaOH via a hydrothermal method. CuO was loaded onto the surface of NaTaO_3_ as a cocatalyst by successive impregnation and calcination. The obtained photocatalysts were characterized by XRD, SEM, UV–vis, EDS and XPS and used to photocatalytically reduce CO_2_ in isopropanol. This worked to both absorb CO_2_ and as a sacrificial reagent to harvest CO_2_ and donate electrons. Methanol and acetone were generated as the reduction product of CO_2_ and the oxidation product of isopropanol, respectively. NaTaO_3_ nanocubes loaded with 2 wt % CuO and synthesized in 2 mol/L NaOH solution showed the best activity. The methanol and acetone yields were 137.48 μmol/(g·h) and 335.93 μmol/(g·h), respectively, after 6 h of irradiation. Such high activity could be attributed to the good crystallinity, morphology and proper amount of CuO loading, which functioned as reductive sites for selective formation of methanol. The reaction mechanism was also proposed and explained by band theory.

## Introduction

Global warming is one of the most major environmental problems that we are facing in the 21st century [1]. Carbon dioxide (CO_2_) contributes significantly to global climate change as it is the main greenhouse gas present in the atmosphere and primarily formed from the consumption of fossil fuels [2]. To date, many methods have been proposed to reduce the emitted CO_2_ concentration. A particularly advantageous approach is the capture of CO_2_ from the atmosphere for the conversion to fuel by using a sustainable source of energy like sunlight. In this way, global warming and energy shortage problems can be solved simultaneously [3–7]. For this purpose, the photocatalytic conversion of CO_2_ to fuel is particularly emphasized.

In 1979, Inoue et al. [8] first reported the photocatalytic reduction of CO_2_ in aqueous solution using several semiconductor materials (WO_3_, TiO_2_, ZnO, CdS, GaP and SiC), producing CH_3_OH, HCOOH, HCHO and trace amounts of CH_4_. In the 1990s, Ta oxide photocatalysts began to draw attention in the field of water splitting. A series of Ta catalysts, such as LiTaO_3_ [9], NaTaO_3_ [10], KTaO_3_ [11], AgTaO_3_ [12], CaTa_2_O_6_ [13], SrTa_2_O_6_ [13], KBa_2_Ta_3_O_10_ [14], were proved to efficiently split water. In the 21st century, the study of Ta catalysts for the reduction of CO_2_ began. Kentaro Teramura et al. [15] prepared ATaO_3_ (A = Li, Na, K) compounds using a solid state reaction (SSR) method to reduce CO_2_ in the presence of H_2_. The only product was CO and the order of photocatalytic activity was LiTaO_3_ > NaTaO_3_ > KTaO_3_, which was consistent with that of the *E*_g_ (band gap) values. However, the highest yield of CO in LiTaO_3_ was 0.42 μmol/g after 24 h of photoirradiation, which was still far from satisfactory. Ye et al. [16] synthesized a series of noble-metal-loaded NaTaO_3_ samples to reduce CO_2_ with water. H_2_ was introduced into this process as an electron donor. Ru/NaTaO_3_ was found to have the best activity (CH_4_ 51.8 μmol/(g·h)) and product selectivity in converting CO_2_ to CH_4_. Junwang Tang and his team [17] prepared KTaO_3_ nanoflakes by a solvothermal method in a hexane–water mixture and reduced CO_2_ using pure water as an electron donor. The activity was quite high for both H_2_ and CO production, achieving 20× (H_2_) and 7× (CO) higher than that of the cubic sample prepared by the solid state reaction. This was an indication that the catalyst morphology played a crucial role in activity. Jeffrey C. S. Wu et al. [18] prepared NiO-loaded InTaO_4_ photocatalysts by a sol–gel method and carried out the photocatalytic reduction of CO_2_ in a self-made optical fiber reactor filled with 0.2 mol/L NaOH solution. The formation rate of methanol was 11.1 μmol/(g·h) under halogen lamp irradiation at 25 °C. Ru-Shi Liu and co-workers [19] prepared a series of nanostructured core–shell materials (Ni@NiO/N-doped InTaO_4_ photocatalysts) for the reduction of CO_2_ to methanol in pure water. In these structures, the core–shell nanostructure might offer a new reaction center transferred from the surface of the InTaO_4_ material.

In this paper, we report the photocatalytic reduction of CO_2_ to methanol using CuO-loaded NaTaO_3_ catalysts. NaTaO_3_ nanocubes were synthesized via a hydrothermal method using Ta_2_O_5_ and NaOH. CuO was loaded onto the surface of NaTaO_3_ by impregnation, where CuO acts as a cocatalyst for CO_2_ reduction, promoting charge transfer and limiting the fast recombination of electrons and holes [20–21]. According to the literature, Cu oxides and Cu cations are active cocatalysts for CO_2_ reduction and could serve as reductive sites for selective reduction of CO_2_ to methanol [22–27]. Isopropanol was employed as both an absorber and a sacrificial reagent due to its good capability to absorb CO_2_ and donate electrons [28–30]. Acetone, an important industrial material, was generated as the oxidation product of isopropanol.

## Experimental

### Catalyst preparation

Tantalum oxide (Ta_2_O_5_, 99.99%), sodium hydroxide (NaOH, 96%) and isopropanol (iPrOH, 99.9%) were purchased from Aladdin Industrial Corporation. Copper nitrate (Cu(NO_3_)_2_·3H_2_O, AR) was purchased from Tianjin Guangfu Chemical Reagent Company. All reagents were used as received without any further purification.

The NaTaO_3_ nanocubes were synthesized by a hydrothermal method as reported by Li et al. [31]. In a typical procedure, 0.442 g of Ta_2_O_5_ and a sufficient amount of NaOH were added into a Teflon-lined autoclave with a total volume of 50 mL, and deionized water was filled up to 40 mL. The autoclave temperature was held at 140 °C for 12 h then cooled to room temperature in air. The obtained product was washed with deionized water several times before being dried at 80 °C in an oven overnight. The as-prepared catalysts were denoted as 1M-NaTaO_3_, 2M-NaTaO_3_, 3M-NaTaO_3_, 4M-NaTaO_3_, corresponding to a NaOH concentration of 1 mol/L, 2 mol/L, 3 mol/L, 4 mol/L, respectively.

CuO was loaded onto the surface of NaTaO_3_ by impregnation. 0.1 g of 2M-NaTaO_3_ and a given amount of Cu(NO_3_)_2_·3H_2_O were mixed in a crucible with 3 mL deionized water. After stirring for 10 min, the crucible was transferred into a muffle furnace and held for 4 h at 450 °C. After cooling down to room temperature, the resulting product was washed and dried at 80 °C overnight. The as-prepared CuO–NaTaO_3_ catalysts were denoted as 1wt-NaTaO_3_, 2wt-NaTaO_3_, 3wt-NaTaO_3_, 4wt-NaTaO_3_ and 5wt-NaTaO_3_ corresponding to 1 wt %, 2 wt %, 3 wt %, 4 wt % and 5 wt % CuO loading on NaTaO_3_, respectively.

### Catalyst characterization

The catalysts were characterized by X-ray diffraction (XRD, Bruke/D8-Advance, Cu Kα radiation, λ = 0.154056 nm) at a scanning rate of 4°/min ranging from 15° to 70°. The morphology was observed with a Hitachi S-4800 field emission scanning electron microscope (SEM) with an accelerating voltage of 3.0 kV. The surface composition of the catalysts was investigated using a Thermo Scientific energy dispersion X-ray (EDX) fluorescence analyzer (with a Mg Kα ADES (*h*ν = 1253.6 eV) source) as an addition to the SEM and XPS (PHA-5400, SPECS, America). Light absorbance was measured with a Shimadzu UV-2550 spectrometer using BaSO_4_ as a reference in the wavelength region of 190–600 nm.

### Photocatalytic reaction

The photocatalytic reduction of carbon dioxide was carried out in a transparent batch reactor with a slurry bed with cooling jacket. The light source was a 250 W high-pressure mercury lamp with an irradiation peak at about 365 nm. The reaction temperature was controlled by a thermostatic water bath at 25 ± 3 °C. The reactor, in which 12 mg of catalyst was dispersed in 12 mL of isopropanol, was tightly sealed during the reaction. A magnetic stirrer agitated at the bottom of the suspension until the reaction ended. Before irradiation, CO_2_ (99.99% purity) was bubbled through the reactor for 30 min to eliminate air and saturate the suspension. A typical run was 6 h.

After reaction, the suspension was centrifuged and the liquid sample was examined by a GC-MS (Agilent 5975C) and quantified by a GC (Agilent 7890A, FID, HP-WAX 60 m column). Control experiments were also carried out to confirm that methanol generation was complete in the CO_2_ reduction. Neither methanol nor acetone was detected in dark or in the absence of catalyst. When N_2_ was bubbled into the reactor instead of CO_2_, only acetone was found after the reaction, indicating the likelihood that the isopropanol was oxidized to acetone.

## Results and Discussions

### Catalyst characterization

**Figure 1:**
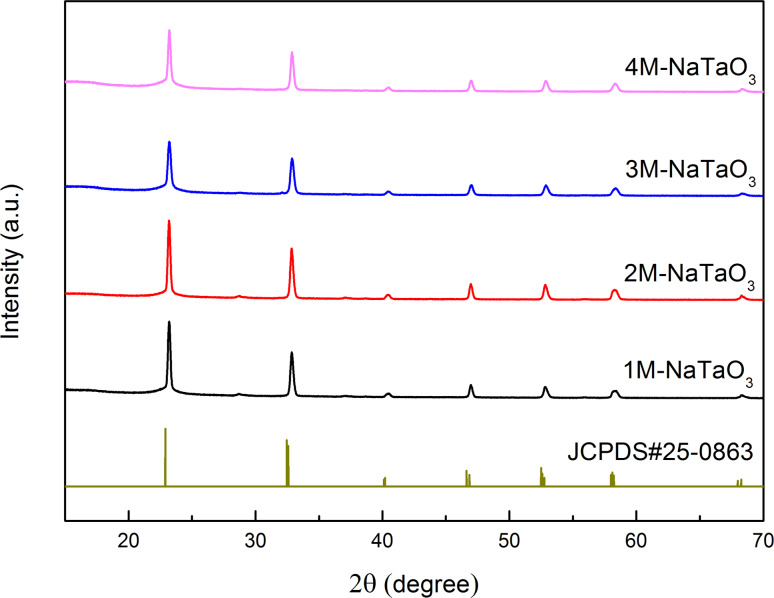
XRD patterns of NaTaO_3_ nanocubes.

Figure 1 shows the XRD patterns of NaTaO_3_ nanocubes prepared with different NaOH concentrations. All diffraction peaks can be indexed to the orthorhombic phase NaTaO_3_ structure according to JCPDS#25-0836 with the space group belonging to I, and lattice parameters *a* = 5.513 Å, *b* = 7.750 Å, and *c* = 5.494 Å. As Ta_2_O_5_ could not completely convert to NaTaO_3_ under conditions of low NaOH concentration during the hydrothermal treatment [32–33], sufficient NaOH was used to ensure that pure NaTaO_3_ was obtained. As calculated by Jade 5.0 software, all the samples had good crystallinity (>98%), which was attributed to the hydrothermal method of catalyst preparation.

**Figure 2:**
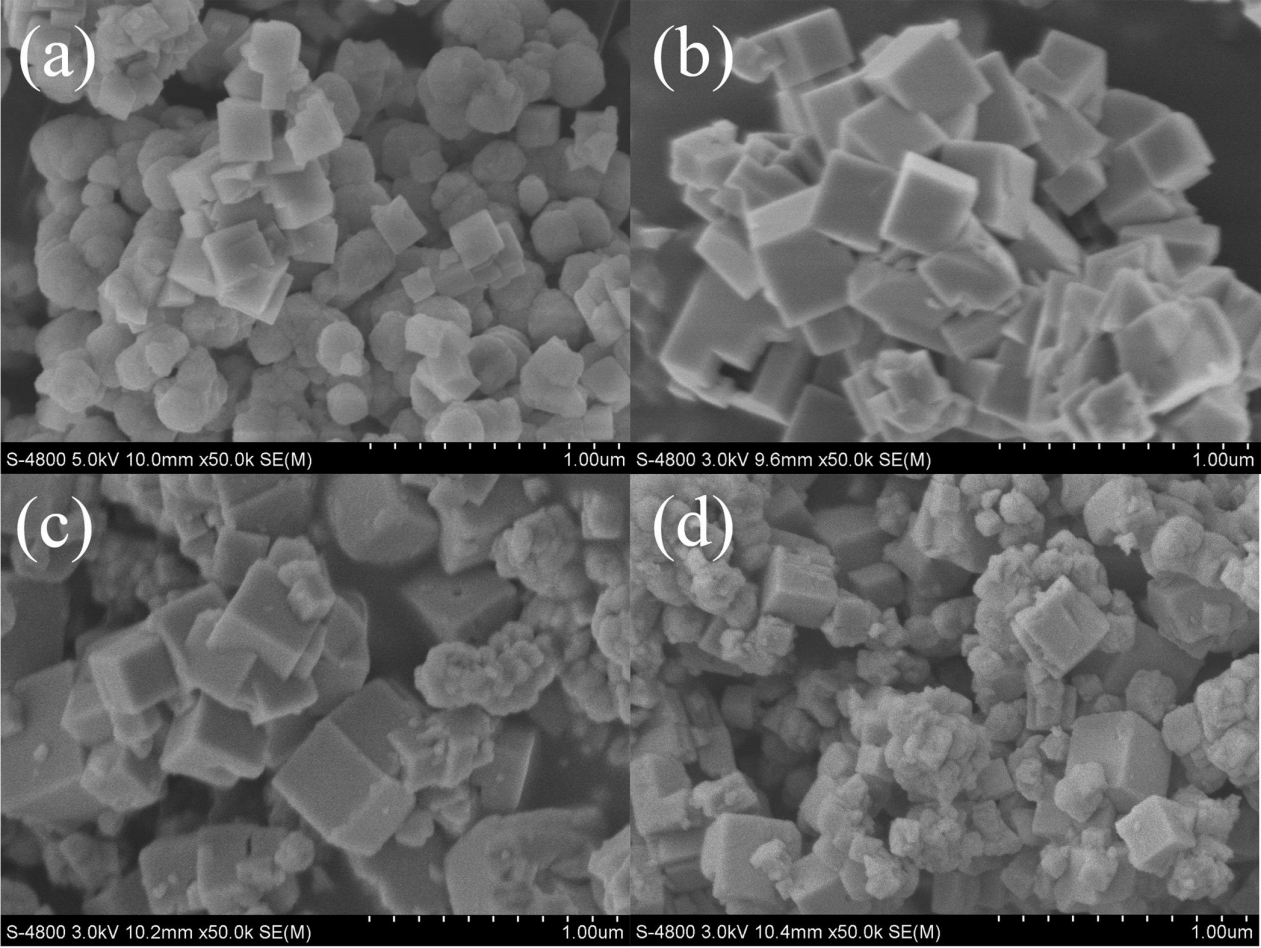
SEM images of NaTaO_3_ nanocubes: (a) 1M-NaTaO_3_, (b) 2M-NaTaO_3_, (c) 3M-NaTaO_3_, and (d) 4M-NaTaO_3_.

Figure 2 shows the SEM images of NaTaO_3_ nanocubes synthesized with different NaOH concentrations. When the NaOH concentration was 1 mol/L, only a small percentage of the NaTaO_3_ grew into cubes. As the NaOH concentration was increased to 2 mol/L, almost all of the particles became larger cubes with an average size of about 300 nm. When the NaOH concentration was increased to 3 mol/L and 4 mol/L, the ideal morphology of the nanocubes was disrupted and fewer nanocubes were observed. Generally, the SEM image of 2M-NaTaO_3_ presents the best morphology. He et al. [34] reported a hydrothermal synthesis of NaTaO_3_ with Ta_2_O_5_ powder and NaOH followed a dissolution–precipitation mechanism, where the concentration of the NaOH solution played a crucial role on the morphology of the crystal. This was confirmed in our work.

**Figure 3:**
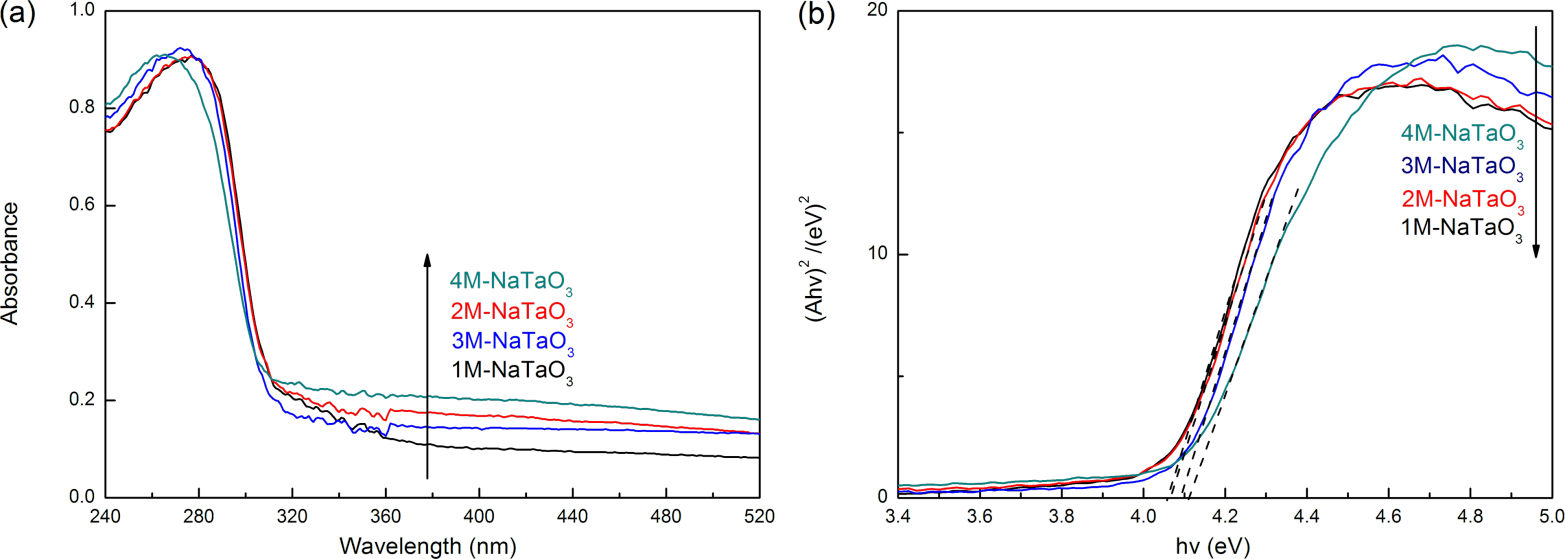
UV–vis diffuse reflectance spectra (a) and optical absorption band edges (b) of NaTaO_3_ nanocubes.

Figure 3 shows UV–vis diffuse reflectance spectra and optical absorption edges of NaTaO_3_ nanocubes prepared with different concentrations of NaOH. From Figure 3a, it can be observed that the main absorption peaks are around 300 nm, which means the powders have an apparent absorption of UV light. The band gap energy (*E*_g_) of each catalyst, prepared with different NaOH concentrations from 1 mol/L to 4 mol/L, can be seen in Figure 3b where the *E*_g_ values of these NaTaO_3_ samples range from 4.06 to 4.12 eV.

**Figure 4:**
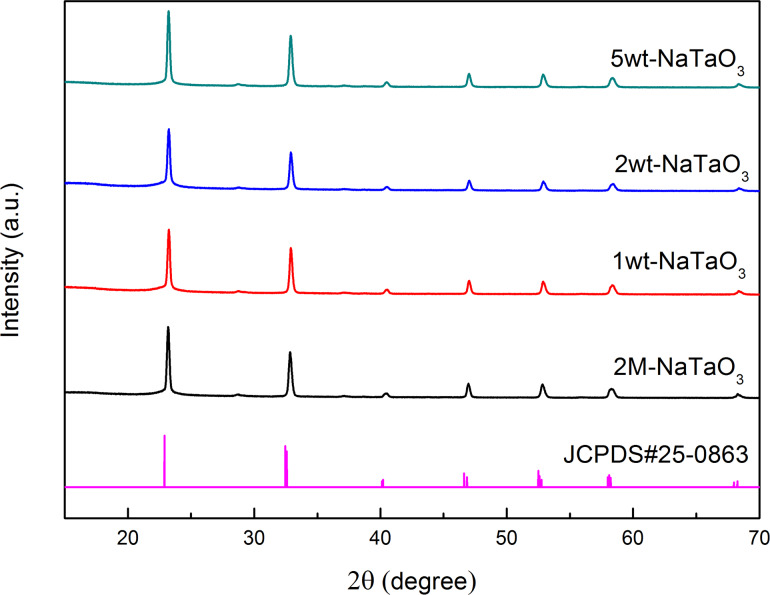
XRD patterns of 2M-NaTaO_3_ nanocubes loaded with different amounts of CuO.

Figure 4 shows XRD patterns of 2M-NaTaO_3_ nanocubes loaded with different amounts of CuO. Comparing with a pure NaTaO_3_ catalyst, the XRD patterns of the CuO-loaded materials seemed not to change, indicating that the crystalline phase of NaTaO_3_ was not affected by CuO loading. CuO was also not detected because the loading amount was relatively low [35].

**Figure 5:**
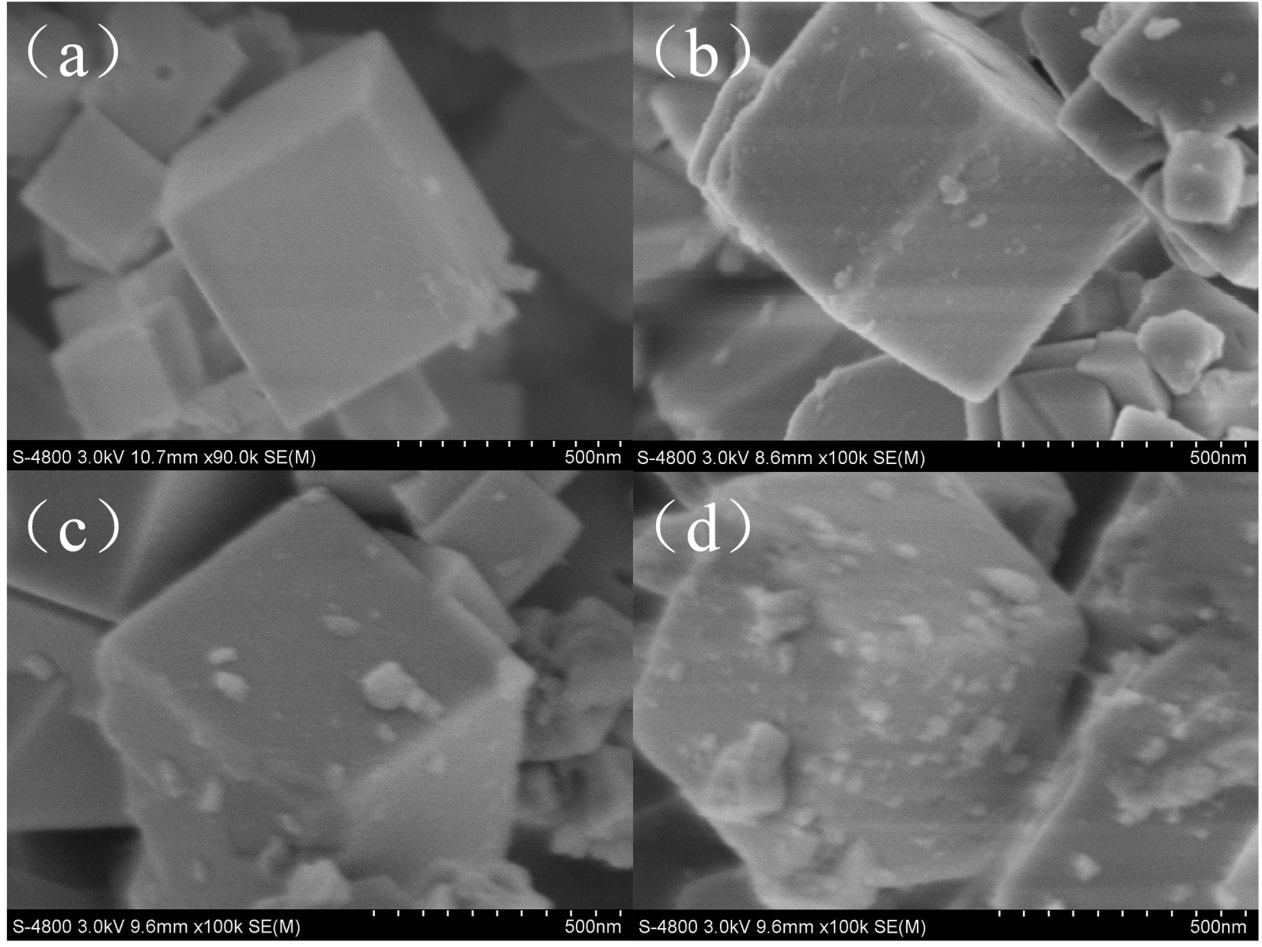
SEM images of CuO–NaTaO_3_ nanocubes: (a) 2M-NaTaO_3_, (b) 1wt-NaTaO_3_, (c) 2wt-NaTaO_3_, and (d) 5wt-NaTaO_3_.

SEM images of CuO–NaTaO_3_ nanocubes are shown in Figure 5. It can be seen that the surface of pure NaTaO_3_ nanocubes was flat and smooth (Figure 5a). With moderate loadings of 1 wt % and 2 wt % CuO, CuO particles were dispersed on the surface of the NaTaO_3_ nanocubes with an average size of tens of nanometers (Figure 5b and Figure 5c). When the loading reached 5 wt %, the CuO nanoparticles began to aggregate and large clusters were formed (Figure 5d).

**Figure 6:**
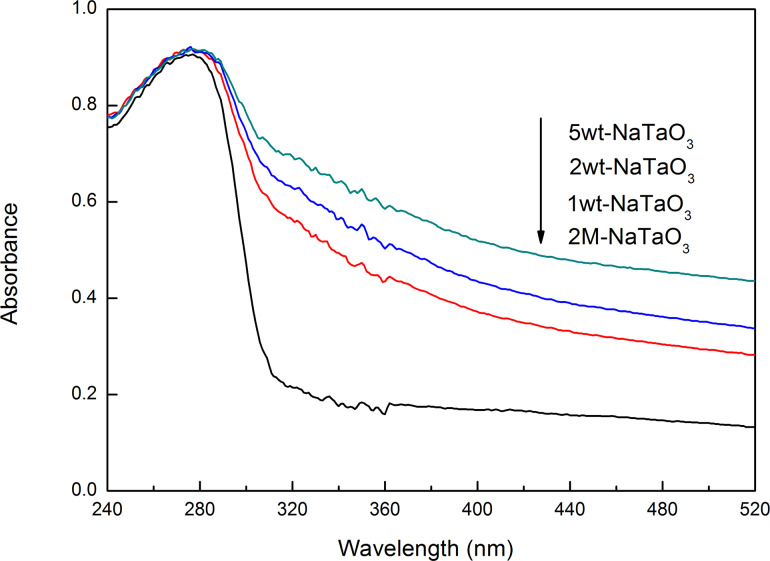
UV–vis diffuse reflectance spectra of CuO-loaded 2M-NaTaO_3_ nanocubes.

The UV–vis diffuse reflectance spectra of CuO–NaTaO_3_ are shown in Figure 6. With a large energy gap, it can be found that pure NaTaO_3_ had low light absorbance in the visible region. After CuO was loaded, the absorbance of the CuO–NaTaO_3_ catalysts in the visible light region (λ > 400 nm) became much stronger with the increase of CuO loading. The increase in visible light absorbance correlates with the formation rate and increase in electrons and holes on the photocatalyst surface [35].

**Figure 7:**
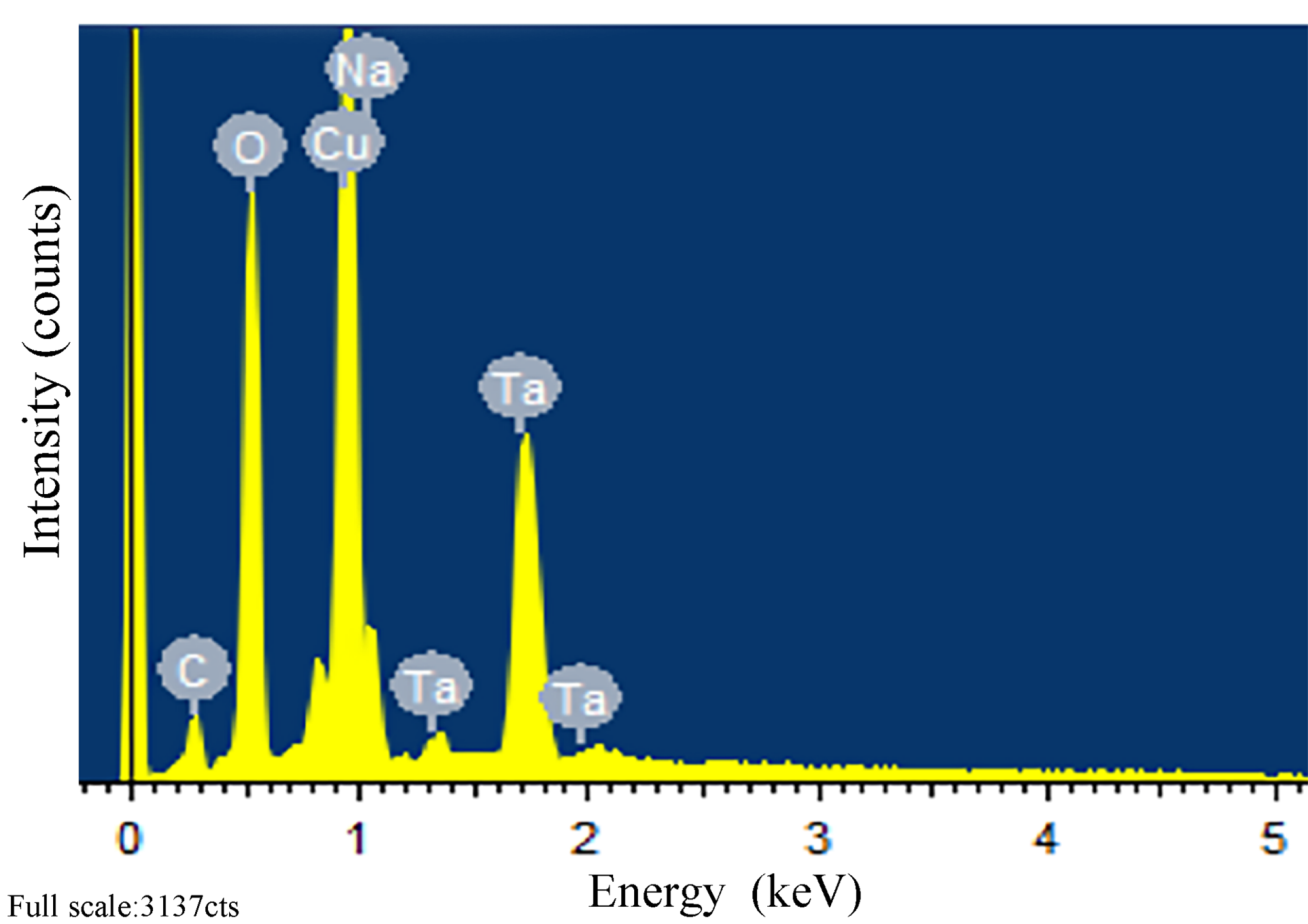
EDS spectrum of 5wt-NaTaO_3_.

**Figure 8:**
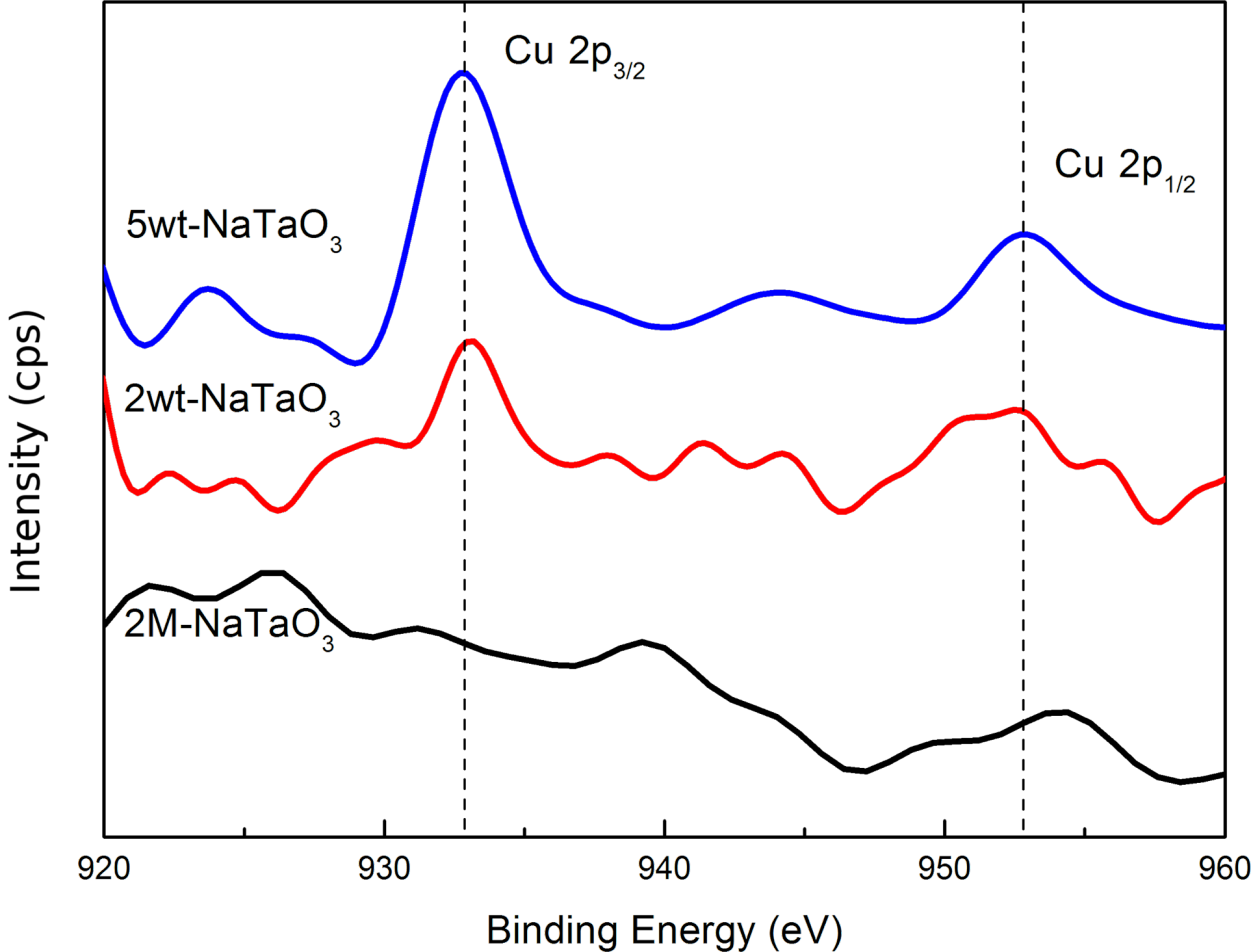
Smoothed Cu 2p XPS peaks 2M-NaTaO_3_, 2wt-NaTaO_3_ and 5wt-NaTaO_3_.

Energy-dispersive X-ray spectroscopy (EDS) and X-ray photoelectron spectroscopy (XPS) were carried out to confirm that CuO was loaded onto the surface of NaTaO_3_ nanocubes. Figure 7 presents the EDS analysis of 5wt-NaTaO_3_, which was performed over a single nanoparticle on the catalyst surface. It can be seen that the main compositional elements of the nanoparticle were Cu and O. Figure 8 demonstrates the Cu 2p XPS peak of 2M-NaTaO_3_, 2wt-NaTaO_3_ and 5wt-NaTaO_3_. The two peaks located at 933.20 eV and 953.20 eV corresponded to Cu 2p_3/2_ and Cu 2p_1/2_ and a satellite peak was also observed at about 944 eV. These peaks were characteristic for Cu^2+^, which indicated that Cu existed in the form of CuO [36–39]. The peak intensity increases with increasing loading.

### Photocatalytic reduction of CO_2_

The photocatalytic activity of CuO–NaTaO_3_ samples was evaluated by photocatalytic reduction of CO_2_ in isopropanol under UV light irradiation for 6 h. Methanol and acetone were generated as the reduction product of CO_2_ and the oxidation product of isopropanol, respectively. 2M-NaTaO_3_ was chosen for CuO loading because of its good morphology. In our experiments, there was no methanol generation in the absence of copper, which was consistent with Hirato’s report [40].

**Figure 9:**
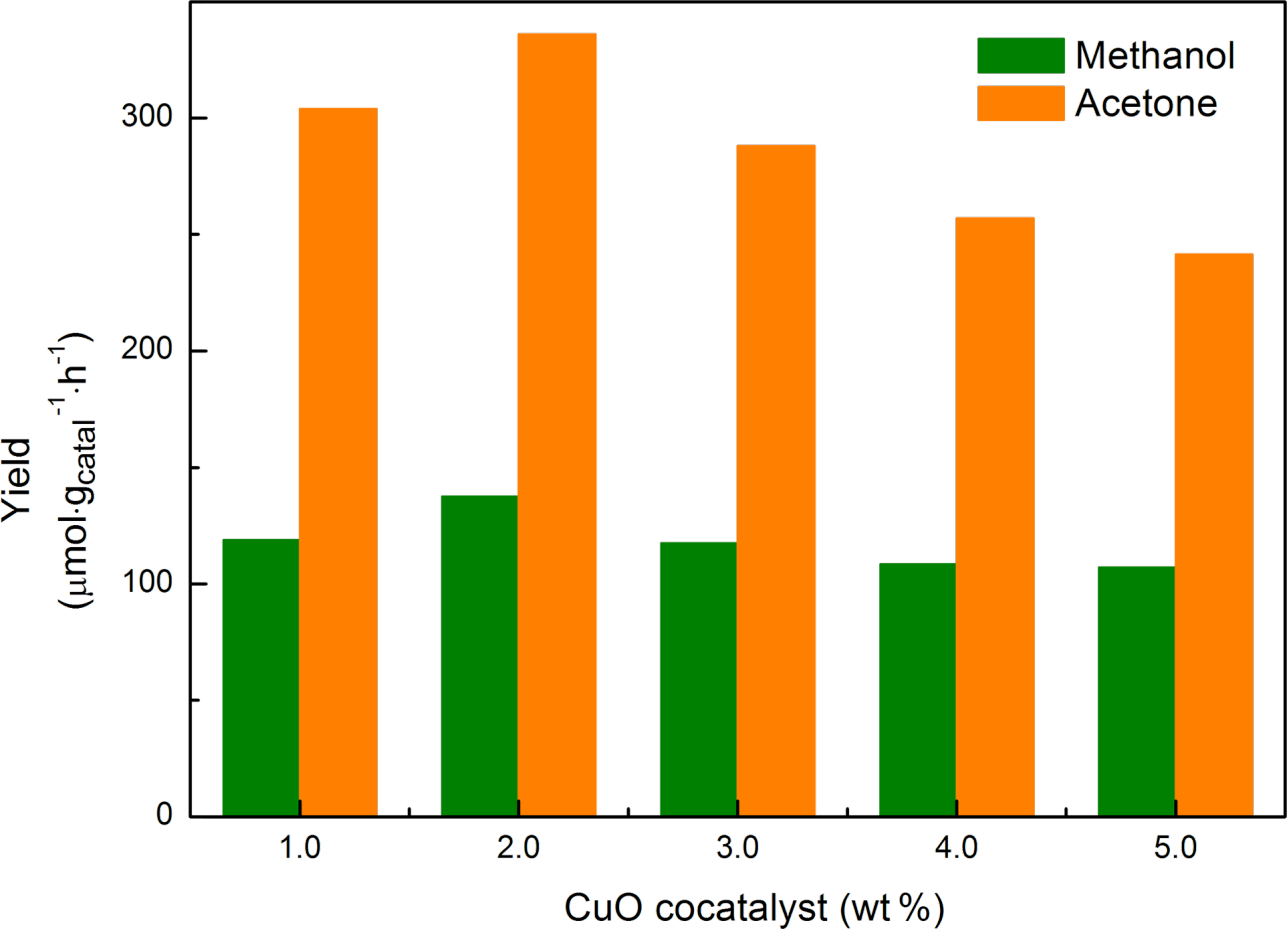
Methanol and acetone yields for 2M-NaTaO_3_ loaded with different amounts of CuO after 6 h of irradiation.

Figure 9 represents the methanol and acetone yield for 2M-NaTaO_3_ loaded with different amounts of CuO after 6 h of irradiation. CuO nanoparticles loaded on the surface of 2M-NaTaO_3_ functioned as reductive sites on which CO_2_ was reduced to methanol. Below the optimal amount of CuO (2 wt %), the activity was promoted with the increase of CuO loading. When the loading was greater than 2 wt %, the activity began to decrease.

In our experiments, 2 wt % CuO loaded 2M-NaTaO_3_ showed the highest activity, which was attributed to its good crystallinity, morphology and proper amount of CuO loading. According to the XRD results of non-loaded NaTaO_3_, all samples had good crystallinity, which was beneficial to photocatalytic activity. The high crystalline quality correlates to a low number of defects. The defects usually function as recombination centers where photogenerated electrons and holes recombine fast, resulting in poor photocatalytic activity [41]. Among these catalysts, 2M-NaTaO_3_ had the best morphology, which played a crucial role in this case. A regular morphology is helpful to the electron transmission process, as it shortens the pathway through which generated electrons transfer from the bulk to the surface of the crystal, thus making the electrons more efficient for the reaction. The CuO loading amount was another important factor. Below the optimal amount of CuO (2 wt %), the activity increased with the increase of CuO loading. For CuO loading greater than 2 wt %, the activity decreased with increasing CuO loading. An explanation for this is that the CuO nanoparticles provide more reductive sites and could reduce the recombination of photogenerated electron–hole pairs with an increase in (moderate) loading, as shown in Figure 5b and Figure 5c [42]. When excessive CuO was loaded, the CuO nanoparticles aggregated to form larger ones (shown in Figure 5d), which decreases the number of effective reductive sites. On the other hand, the large CuO particles could also decrease the efficient separation of electron–hole pairs as compared with smaller ones. Both of these situations could lead to a poor activity.

### Reaction mechanism

**Figure 10:**
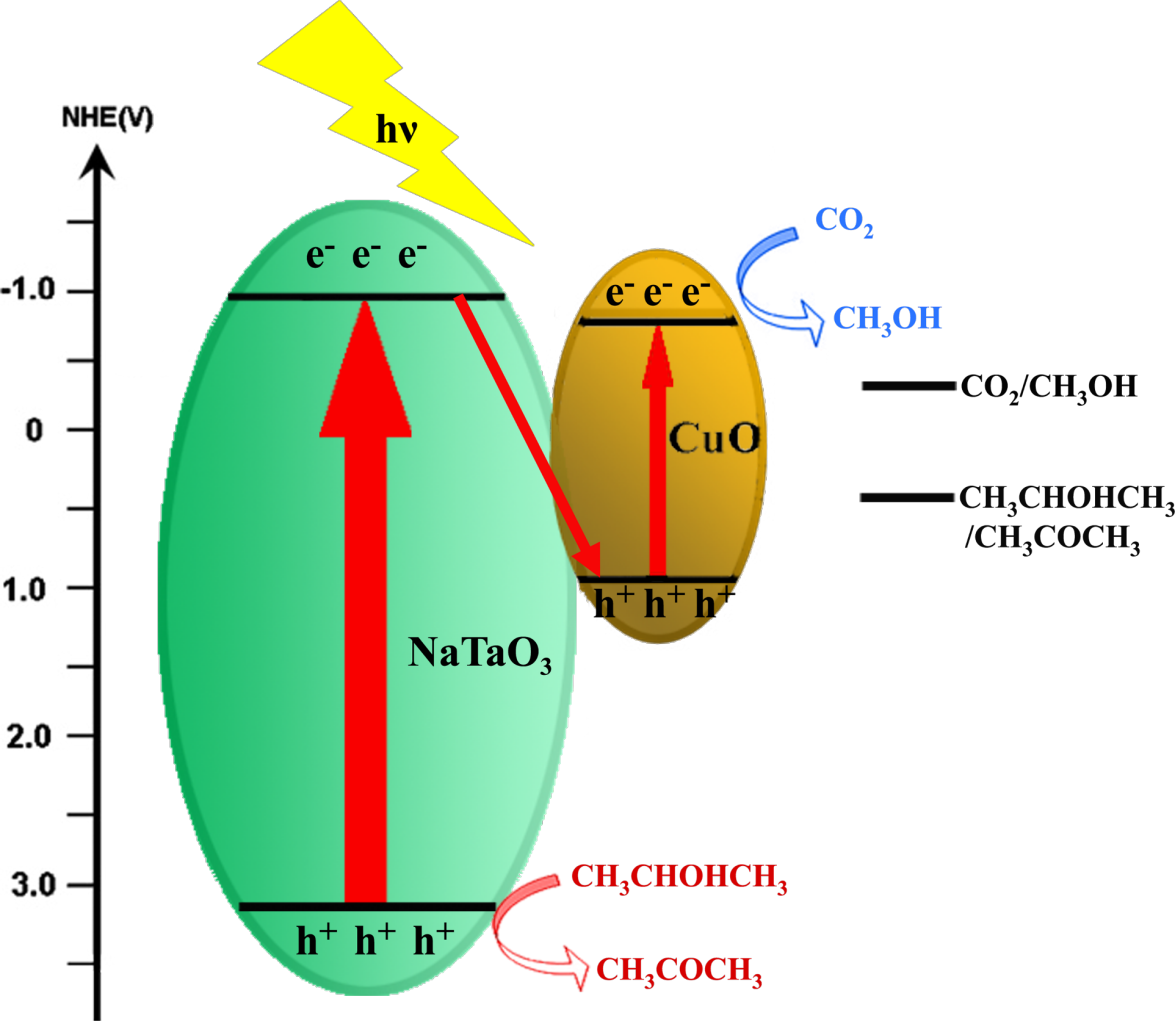
Schematic diagram for photocatalytic reduction of CO_2_ to methanol in CuO–NaTaO_3_ photocatalyst under UV light irradiation.

The mechanism for photocatalytic reduction of CO_2_ to methanol in isopropanol is shown in Figure 10. When the CuO–NaTaO_3_ catalysts were irradiated by UV light, photogenerated electron–hole pairs were created. The electrons in the conduction band (CB) of NaTaO_3_ (*E*_CB_ = −0.92 V vs NHE at pH 7, the same below) [41] could recombine with holes in the valence band (VB) of CuO, enhancing the separation and prolonging the lifespan of photogenerated electron–hole pairs. It was believed that the reduction reaction happened in the CB of CuO (−0.78 V) [35], where CO_2_ reacted with electrons and protons to generate methanol (*E*_CO2/CH3OH_ = −0.38 V) [43], as no methanol was detected using pure NaTaO_3_. The valence band (VB) potential of NaTaO_3_ is 3.13 V, which is more positive than the potential of isopropanol oxidation to acetone (about 0.47 V) [44–45], thus the oxidation reaction could happen in the VB of NaTaO_3_.

The reaction in the CB of CuO was as follows:


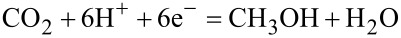


The isopropanol was oxidized into acetone and protons by holes in the valence band of NaTaO_3_, which was illustrated by G. R. Dey [46]:


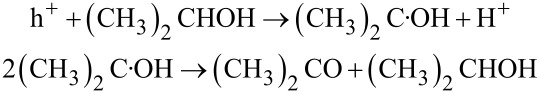


and the overall reaction was


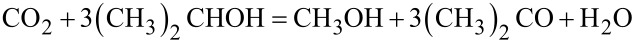


Theoretically, one mole of methanol and three moles of acetone were generated simultaneously. But in our experiments, the mole ratio of acetone to methanol ranged from 2.33 to 2.55. This was attributed to the generation of acetone that was sequentially oxidized into small molecules, which were not detectable by GC.

## Conclusion

NaTaO_3_ nanocubes were synthesized with Ta_2_O_5_ powder and NaOH solution via a hydrothermal method. CuO was loaded onto the surface of NaTaO_3_ by impregnation to suppress the electron–hole recombination and functioned as a reductive site for methanol formation. Acetone was also generated as the oxidation product of isopropanol. With 2 wt % CuO loading, NaTaO_3_ prepared by 2 mol/L NaOH solution showed the best performance. The highest yields of methanol and acetone were 137.48 μmol/(g·h) and 335.93 μmol/(g·h), respectively, after 6 h of irradiation. These good yields were attributed to the good crystallinity and morphology of NaTaO_3_ and the proper loading amount of CuO on NaTaO_3_. The mechanism for photocatalytic reduction of CO_2_ in isopropanol to methanol was also proposed and explained by band theory.
